# Successful treatment of glandular tularemia with azithromycin in a pregnant woman in Austria

**DOI:** 10.1007/s15010-023-02160-2

**Published:** 2024-01-11

**Authors:** Lorenz Schubert, Marita Koelz, Manuel Kussmann, Sylvia Metz-Schimmerl, Selma Tobudic, Ludwig Traby, Matthias G. Vossen, Stefan Winkler

**Affiliations:** 1 Division of Infectious Diseases and Tropical Medicine, Department of Internal Medicine I, Medical University of Vienna, Vienna, Austria; 2 Clinical Institute of Pathology, Medical University of Vienna, Vienna, Austria; 3 Department of Radiology, Medical University of Vienna, Vienna, Austria

**Keywords:** Tularemia, Pregnancy, Macrolides

## Abstract

Treatment of tularemia during pregnancy is challenging due to toxicity of standard treatment regimens. Here, we report a 31-year-old woman with glandular tularemia who was successfully treated with intravenous azithromycin. Follow-up examinations over a 6-month period showed a sustained response to treatment. She later gave birth to a healthy child.

## Introduction

*Francisella tularensis*, an aerobic, fastidious Gram-negative bacteria, is the causative agent of human tularemia. In general, the majority of infections are caused by *F. tularensis subspecies tularensis*, the more virulent subspecies, and *F. tularensis subspecies holarctica*, the less virulent subspecies. Both subspecies vary in the geographic distribution, *F. tularensis subspecies tularensis* is responsible for the majority of cases in North America, while *F. tularensis subspecies holarctica* causes most of the tularemia in Europe. The European Center of Disease Prevention and Control reported 876 cases of human tularemia in 2021 [1]. Treatment strategies comprise quinolones, tetracyclines, and aminoglycosides. However, these agents are generally to be avoided in specific cohorts such as children and during pregnancy due to toxicity issues [2]. Clinical experience regarding alternative agents, such as macrolides, is scarce.

## Case report

Here, were report the case of a 31-year-old pregnant woman (pregnancy week 20) who was admitted to the outpatient clinic of the Division of Infectious Diseases and Tropical Medicine of the Medical University of Vienna. She reported persisting fever, shivering, fatigue, and neck and joint pain lasting for nearly a month. She already had received penicillin V, initiated by a general practitioner who suspected a bacterial tonsillitis, as well as intravenous amoxicillin/clavulanic acid, which she received in a hospital after worsening of symptoms. Ultrasound examination revealed enlarged lymph nodes, the largest of which was 4.3 × 1.7 cm, and the patient was referred to our center for further evaluation.

**Fig. 1:**
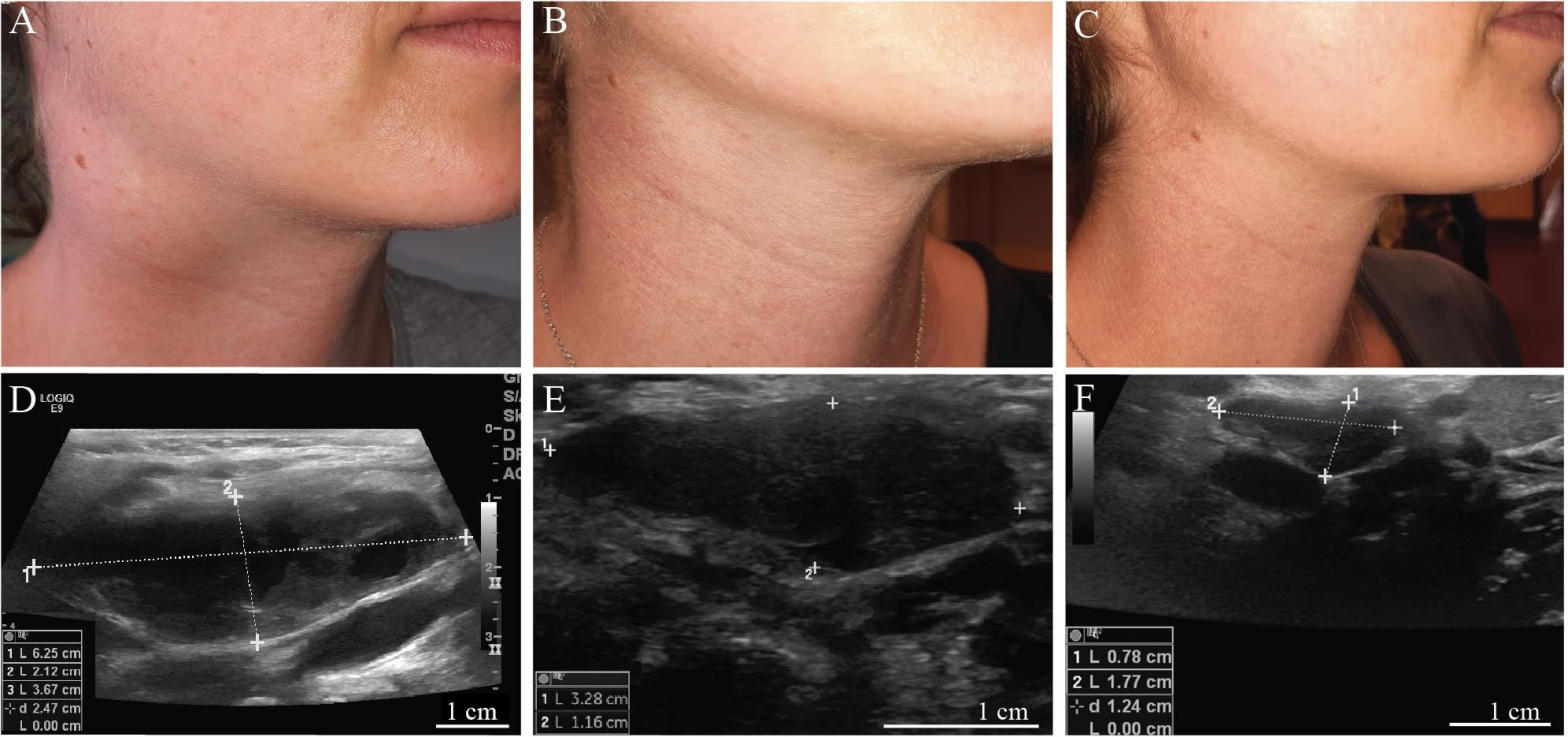
The clinical images as well as ultrasound controls at the day of admission (**A**, **D**), after 1 month of treatment (**B**, **E**) and after 6 months (**C**, **F**)

**Fig. 2:**
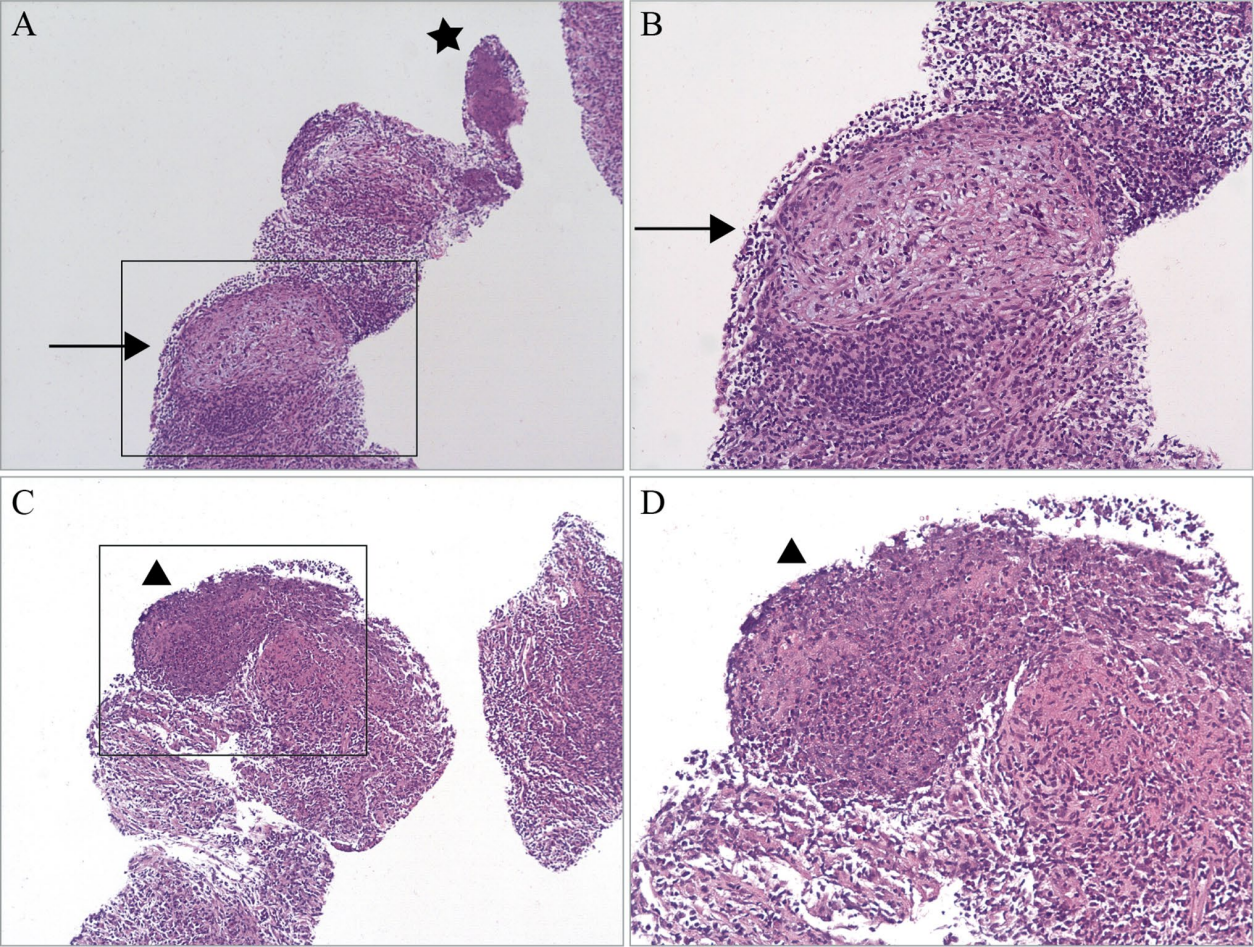
**A** Partially necrotizing epithelioid cell granulomas (arrow) and abscessing lymphadenitis (star). **B** The granuloma (arrow) in a higher amplification. **C** An abscess forming inflammation (triangle). **D** The abscess forming inflammation (triangle) in higher amplification

At admission, the clinical examination revealed significant right sided submandibular swelling. Inspection of the mouth did not demonstrate signs for tonsillitis, mucositis, or any other focal inflammation. Subsequent ultrasonography showed that the lymph nodes were increased in size (largest lymph node 6.3 × 2.1 × 3.7 cm) with central necrosis and hypervascularization (Fig. 1). For further diagnosis, a biopsy was performed. Histological analysis demonstrated an abscess-forming lymphadenitis, with formation of partially necrotizing granulomas (Fig. 2).

Since the patient lived on a farm and had regular contact with animals, glandular tularemia was suspected and another biopsy and serological analysis were performed. The enzyme immunoassay demonstrated *F. tularensis* IgG-antibody (196 U/ml, negative < 10U/ml), as well as *F. tularensis* IgM-AK (> 400 U/ml, negative < 10 U/ml). *F. tularensis* was later confirmed by polymerase chain reaction out of the lymph node tissue.

Due to safety concerns regarding aminoglycosides, tetracyclines and quinolones during pregnancy, antimicrobial treatment with azithromycin was initiated. Azithromycin was administered intravenously every day (500 mg) over 3 days to achieve adequate tissue levels. Interestingly, immediately after the first administration of azithromycin, the patient experienced an episode of fever and more pronounced pain of the affected lymph nodes. The fever resolved rapidly and was gradually followed by an improvement in her general condition. The azithromycin regimen was repeated every week for 1 month. No azithromycin-related adverse events were reported. Regularly performed ultrasounds of the lymph node demonstrated an impressive response to therapy (ultrasound follow-up 1 week: 5.4 × 2 × 3.6 cm; follow-up 1 month: 4.3 × 1.6 × 3.3 cm, follow-up 6 months: 2.7 × 1 × 1.8 cm) (Fig. 1). Gynecological controls for pregnancy revealed no abnormalities and the patient gave birth without complications. No additional antimicrobial treatment or surgical intervention was needed after pregnancy. Table 1 shows the results of serological tests performed during and after therapy.

**Table 1:** Demonstrates results for the agglutination test, as well as for the IgM and IgG specific enzyme immunoassay (EIA)

Days	Agglutinations test	EIA IgM	EIA IgG
1	1:1280	> 400	159
8	1:5000	> 400	196
35	1:640	391	148
91	1:640	> 400	135
189	1:320	> 400	> 300

## Discussion

*F. tularensis* during pregnancy poses a risk to pregnant women and the fetus*.* Intrauterine death caused by *F. tularensis* is well described in animals, and discussed in humans [3, 4]. Most frequently used regimens during pregnancy include aminoglycosides or aminoglycoside and quinolone combinations [5, 6]. Both options are potentially toxic during pregnancy and should be avoided if possible [2]. The effectiveness of better tolerable agents, such as macrolides, is discussed in vitro, but differs dependent on the subspecies. Whereas, *F. tularensis subspecies tularensis* (type A strain) (minimum inhibitory concentration (MIC) 0.125–2 mg/L) and *F. tularensis subspecies holarctica* (type B strain) *biovar I* (MIC 0.064–2 mg/L) are generally susceptible to macrolides, *F. tularensis subspecies holarctica biovar II* is generally not susceptible (MIC > 256 mg/L) [7]. The erythromycin resistance in *F. tularensis* can be linked to a gene mutation, which was only found in the phylogenetic group B12 [8]. Phylogeography analysis of *F. tularensis* in Europe demonstrated that most infections in Central and Eastern Europe are caused by the macrolide-resistant group, whereas *F. tularensis* infections in Western Europe are mainly caused by the macrolide-susceptible group [9].

Previously, one pregnant patient with tularemia caused by a *F. tularensis type A strain* in North America, as well as one patient with tularemia caused by *F. tularensis subsp. holarctica* *biovar 1* strain in France, was successfully treated by azithromycin [10, 11]. In another report in France, two other pregnant tularemia patients were successfully treated with josamycin or azithromycin, but only improved after surgical removal of suppurated lymph node. *F. tularensis* subspecies was not specified in this report [12].

In Austria, phylogenetic analysis demonstrated that most infections are caused by a macrolide-resistant subspecies (*F. tularensis subsp. holarctica* clade B.13) [9]. We could not determine the subspecies of *F. tularensis* in this patient, because it did not grow in culture and therefore insufficient material for classification was available. However, just after the first treatment cycle, the patient experienced fever as well as temporary increase of swelling of the lymph nodes after the first dose of azithromycin, which was surprising and might have indicated some kind of Jarisch–Herxheimer like reaction due to the rapid destruction of bacteria [13]. This treatment related inflammatory response as well as the rapid improvement afterward strengthened our decision to continue the treatment with azithromycin.

We preferred intravenous azithromycin over other macrolides due to the longer half-life, higher volume of distribution, and interstitial fluid-plasma concentrations, allowing outpatient parenteral antibiotic treatment [14]. To achieve higher tissue concentrations, azithromycin was administered intravenously, and repeated over 3 days. In a retrospective analysis, 15% of the tularemia patients treated with doxycycline (≥ 14 days) experienced treatment failure [15]. Hence, we decided to continue treatment four times over a period of 4 weeks to prevent relapse and subsequent surgery. To ensure response to treatment and to allow adjustment of treatment as early as possible, the patient was monitored closely. Clinical examinations and ultrasound assessments demonstrated a rapid and sustained response. She later gave birth to her child without complications.

Overall, this case report supports azithromycin as a therapeutic option for tularemia in patients during pregnancy. Pretreatment evaluation of *F. tularensis* subspecies may nevertheless be important, especially in areas with a high prevalence of azithromycin-resistant subspecies.
